# Breathing Exercise for Chronic Pain Management in Breast Cancer Survivors: Feasibility Outcomes and Qualitative Insights from a Pilot Randomised Controlled Trial

**DOI:** 10.3390/healthcare14050641

**Published:** 2026-03-03

**Authors:** Haiying Wang, Jing-Yu (Benjamin) Tan, Li-Qun Yao, Tao Wang

**Affiliations:** 1 School of Nursing and Midwifery, University of Southern Queensland, Ipswich, QLD 4305, Australia; emily.wang@unisq.edu.au (H.W.); benjamin.tan@nd.edu.au (J.-Y.T.); 2 Centre for Health Research, University of Southern Queensland, Springfield, QLD 4300, Australia; 3 Institute for Health, School of Nursing and Midwifery, Charles Darwin University, Darwin, NT 0810, Australia; 2013039@fjtcm.edu.cn; 4 School of Nursing and Midwifery, The University of Notre Dame Australia, Fremantle, WA 6160, Australia; 5 College of Nursing, Fujian University of Traditional Chinese Medicine, Fuzhou 350122, China

**Keywords:** feasibility study, pilot RCT, breathing exercise, cancer care

## Abstract

**Background:** Feasibility studies enable the refinement of complex interventions and procedures prior to definitive trials. To establish robust evidence for clinical applications, this study evaluated the feasibility, acceptability and safety of an evidence-based breathing exercise (BE) intervention protocol among breast cancer survivors experiencing chronic pain. **Methods:** In an open-label pilot randomised controlled trial with a nested qualitative evaluation, 72 women were randomised into a BE plus routine care group (*n* = 36) or a routine care alone group (*n* = 36). Feasibility and acceptability outcomes included recruitment, retention, instrument completeness and suitability, intervention adherence, safety, and participants’ perceived experience of practicing BE and participating in this study. Outcome assessments occurred at baseline (week 0), post-intervention (week 5), and follow-up (week 9). Quantitative data were analysed descriptively. Qualitative interviews (*n* = 20) explored participant experiences, and the data were analysed thematically. **Results:** The feasibility was high, with an 84% recruitment (72/86) and 94% retention (68/72), and the recruitment process took 19 weeks. The overall BE intervention adherence was 82.4%. Questionnaire completion was satisfactory, with minimal missing values reported in the questionnaire. No serious adverse events occurred. Evidence from qualitative evaluation reinforced the feasibility from quantitative data. **Conclusions:** The BE protocol and study procedures were feasible, acceptable, and safe in this population. A fully powered RCT is warranted to determine the effectiveness and durability of the outcomes.

## 1. Introduction

Breast cancer survival has improved substantially over recent decades; however, many women continue to experience long-term treatment-related sequelae. Chronic pain is among the most prevalent and burdensome of these consequences [1,2,3], with well-documented adverse effects on physical functioning, emotional well-being, and overall quality of life [3,4].

Pharmacological therapies remain central to cancer-related pain management but are frequently associated with side effects, safety concerns, and limited acceptability [1,2,3]. Consequently, there is a growing interest in simple, low-cost, non-pharmacological interventions that can complement standard care and support patient self-management.

Breathing exercises (BEs) are widely used healthcare interventions with a long history of promoting health and well-being, particularly in Asian countries [5,6]. Emerging evidence suggests that BEs may modulate autonomic nervous system activity, promote relaxation, and reduce pain intensity [7]. Primary studies have demonstrated the promising effects of BEs in alleviating pain among cancer patients [8,9]. However, the current evidence base is methodologically limited, characterised by small sample sizes, heterogeneous intervention protocols, inconsistent reporting, and a lack of data on feasibility, acceptability, and safety [8]. Robust evidence is therefore required to determine its effectiveness.

Guided by a systematic review conducted by our research team, an extensive literature review, and consultation with clinical experts, we developed an evidence-based BE intervention protocol specifically designed for chronic pain management in breast cancer survivors [7]. To evaluate this protocol, we conducted a pilot randomised controlled trial (RCT) with a nested qualitative evaluation to assess intervention safety and to examine the feasibility of key methodological procedures prior to undertaking a definitive RCT. This approach aligns with the Medical Research Council (MRC) Framework for developing and evaluating complex interventions, with the explicit aim of optimising study design, refining intervention delivery, and avoiding the unnecessary use of research resources [10,11].

The aim of this paper is to report the feasibility, acceptability, safety, and procedural integrity of a structured BE intervention protocol in a pilot RCT for breast cancer survivors to manage chronic pain. This manuscript focuses on feasibility outcomes only; preliminary clinical outcomes have been reported elsewhere [12].

## 2. Methods

The study comprised two sequential phases: an open-label pilot RCT, followed by an embedded qualitative process evaluation. The study was conducted in a tertiary hospital in Sichuan Province, China, prospectively registered (NCT05257876).

### 2.1. Pilot RCT Study Design

#### 2.1.1. Participants

Eligible participants were women aged ≥18 years with stage I–IIIa breast cancer, experiencing persistent or intermittent pain ≥ 3 months with average pain ≥ 4/10 in the past week, and having completed primary treatment ≥ 3 months prior. Exclusions included severe physical limitations preventing BE practice, cognitive impairment, planned treatments likely to influence pain, concurrent non-pharmacological pain interventions, and pre-existing chronic pain predating cancer diagnosis. A total of 72 participants were recruited, with 36 participants per group. As this was a pilot RCT primarily designed to assess feasibility and acceptability, a power-based sample size calculation was not undertaken. The sample size rationale has been reported in the published study protocol [4], following feasibility trial guidance, and targeted 30 participants per group, allowing 20% anticipated attrition (36 participants per group recruited). More details can be found in previous publications [4,12].

#### 2.1.2. Randomisation and Allocation

All participants were randomised after the baseline assessment completion. Participants were randomised 1:1 using an independent online service with permuted blocks (2, 4, 6). Allocation concealment was maintained until enrolment [4,12].

#### 2.1.3. Intervention

All participants received standard care and a pain information booklet (refer to Supplementary Materials for standard care). The intervention group additionally practised a structured BE protocol (slow pursed-lip breathing; inspiration-to-expiration ratio 1:2–3), with 3–5 sessions/day, 5 min/session, for 4 weeks. Training was delivered by nurses with video support; participants’ competency of practicing BE was confirmed after training. Control participants were offered post-trial BE training after final data collection. Details of intervention development and implementation can be found in the previous publications [4,12].

#### 2.1.4. Procedures and Time Points

Participants were recruited via clinician referral and self-referral. After consent and baseline assessment, intervention participants commenced BE and recorded sessions in daily logbooks. Weekly phone contact with all participants was conducted by research assistants to support adherence, monitor side effects, and record treatment changes. Assessments occurred at baseline (week 0), post-intervention (week 5), and follow-up (week 9) [4,12]. Participants who did not attend scheduled follow-up assessments were contacted via telephone up to three times over a two-week period to encourage completion and understand reasons for non-attendance.

### 2.2. Feasibility Assessment

#### 2.2.1. Feasibility of Participants Recruitment

The feasibility of participant recruitment was evaluated using the following indicators: (1) referral rate: the proportion of referrals made by clinicians relative to all referrals received from inpatient and outpatient clinics; (2) recruitment rate: the proportion of eligible individuals who consented to participate in the study; (3) retention rate: the proportion of participants who completed the study through to follow-up assessment; (4) dropout rate: the proportion of participants who discontinued participation after randomisation; (5) the time required to recruit the target sample size; and (6) feedback from participants who withdrew, including their reasons for discontinuation [4,12].

#### 2.2.2. Feasibility of Study Questionnaires

The feasibility of the questionnaires was assessed by calculating missing responses at both the item and scale levels for the Brief Pain Inventory (BPI), Hospital Anxiety and Depression Scale (HADS), Quality of Life Cancer Survivors Version (QOL-CSV), and Functional Assessment of Cancer Therapy—Breast (FACT-B) at baseline (Time point 1, week 0), post-intervention (Time point 2, week 5), and follow-up (Time point 3, week 9). Item-level missing data referred to the percentage of unanswered items, and scale-level missing data referred to the percentage of questionnaires with at least one missing item. The time required to complete each questionnaire was also recorded from participant reports [4,12].

#### 2.2.3. Feasibility and Acceptability of the BE Intervention

The feasibility and acceptability of the intervention were evaluated using the following measures: (1) adherence rate: the proportion of breathing exercise (BE) sessions completed by participants relative to the prescribed number; the prescribed dose was 3–5 sessions per day (we took 3 sessions as the minimum for calculation), 7 days per week, for 4 weeks (84 sessions total); adherence was calculated as (Total sessions completed by all participants) ÷ (Total prescribed sessions [84 sessions × 36 participants]) × 100; individual adherence was classified using established benchmarks [13]: >80% completion indicated acceptable adherence, and ≥95% indicated optimal adherence; (2) participant feedback: collected through feedback forms completed by those in the intervention group; and (3) safety: monitored through participants’ BE logbooks and supplemented by information obtained during weekly telephone contacts. These outcomes were assessed throughout the intervention period and again immediately after its completion at week 5 (Time point 2) [4,12].

#### 2.2.4. Data Analysis

Descriptive statistics were used to assess the feasibility of the pilot RCT in terms of participant recruitment, suitability of the assessment instruments, and acceptability of the BE intervention protocol. This pilot RCT was not powered for efficacy evaluation; and its analysis was primarily descriptive per the CONSORT extension for pilot/feasibility assessment [14].

#### 2.2.5. Safety Monitoring

Adverse events (AEs) were defined according to the Common Terminology Criteria for Adverse Events (CTCAE) Version 5.0 [15] as any untoward medical occurrence during the study period, regardless of causality. Serious adverse events (SAEs) were defined as events that were life-threatening, required hospitalisation, resulted in persistent disability, or were fatal. AEs were systematically solicited through two methods: (1) participant self-report via daily logbooks, and (2) active solicitation during weekly telephone calls with research assistants throughout the 4-week intervention period. All reported events were graded using CTCAE Version 5.0 criteria: Grade 1 (mild: minimal symptoms, not interfering with daily activities), Grade 2 (moderate: bothersome symptoms interfering with some activities), or Grade 3 (severe: medically significant, requiring intervention). Events were assessed for relatedness to the intervention using standard attribution categories: related, possibly related, or unrelated.

#### 2.2.6. Progression Criteria

Following the framework recommended by Mellor, Albury [16], we applied context-specific progression criteria post hoc to evaluate whether this pilot trial supports advancement to a definitive RCT. Progression benchmarks were developed based on: (1) recruitment and retention requirements for cancer trials; (2) adherence thresholds for self-administered interventions; (3) data completeness needs for self-report outcomes; and (4) expected safety profile for low-risk breathing exercises. Each feasibility domain was classified using a traffic-light system: Green (Go: proceed to definitive trial), Amber (Amend: proceed with modifications), or Red (Stop: do not proceed). The specific benchmarks and rationale for each domain are presented in the Results (Section 3.1.6).

### 2.3. Qualitative Process Evaluation

The qualitative process evaluation was designed to assess the feasibility and acceptability of the intervention and trial procedures, consistent with MRC guidance for process evaluation in feasibility studies [17]. The focus was on understanding barriers and facilitators to implementation, appropriateness of study procedures, and participant experiences that could inform refinement of the definitive trial.

#### 2.3.1. Participants

Participants for the qualitative process evaluation were selected from those who had participated in the pilot RCT. A purposive sampling strategy was used to capture variation across several factors: baseline expectations of BE effectiveness (high/low), self-reported pain relief after four weeks (yes/no), and levels of satisfaction with the intervention (yes/no). The final sample size was guided by the principle of data saturation, whereby recruitment ceased once no new insights emerged.

#### 2.3.2. Procedure

Semi-structured interviews were conducted by the researcher and two trained research assistants. The assistants underwent a structured five-phase training program covering ethics, qualitative methods, and mock interviews to ensure data quality [18]. While the researcher led most interviews, participants who spoke local dialect not understood by the researcher were interviewed by research assistants fluent in that dialect. All interviewers had been actively involved in the project and were familiar with the intervention. With consent, interviews were audio-recorded, transcribed verbatim, and checked for accuracy by the interviewees and research team members. An interview guide, informed by a literature review, provided key questions and prompts to encourage open discussions.

#### 2.3.3. Data Analysis

Data were analysed thematically using NVivo 14 following Braun and Clarke’s six-step approach, including transcript familiarisation, coding, searching for themes, reviewing potential themes, defining and naming themes, and reporting [19]. Trustworthiness was ensured through confirmability, credibility, transferability, and dependability [20]. Confirmability was supported by member checking, with participants reviewing transcripts for accuracy, while coding was conducted by the researcher and cross-checked with research team members until consensus was reached. Credibility was enhanced through careful attention to study design and iterative discussions during analysis [21]. Transferability was supported by providing a detailed account of procedures and findings, and dependability was established by transparent reporting of the study design, analysis steps, and synthesis of results [22].

### 2.4. Ethical Approval

Ethical approval for the study was obtained from the Human Research Ethics Committees at Charles Darwin University (H21089) and the Affiliated Hospital of Southwest Medical University (KY2022107). All participants were fully informed about the purpose of the study, potential benefits and risks, confidentiality safeguards, and their right to withdraw at any time without penalty. Written informed consent was obtained from all participants prior to their participation.

## 3. Results

**Figure 1:**
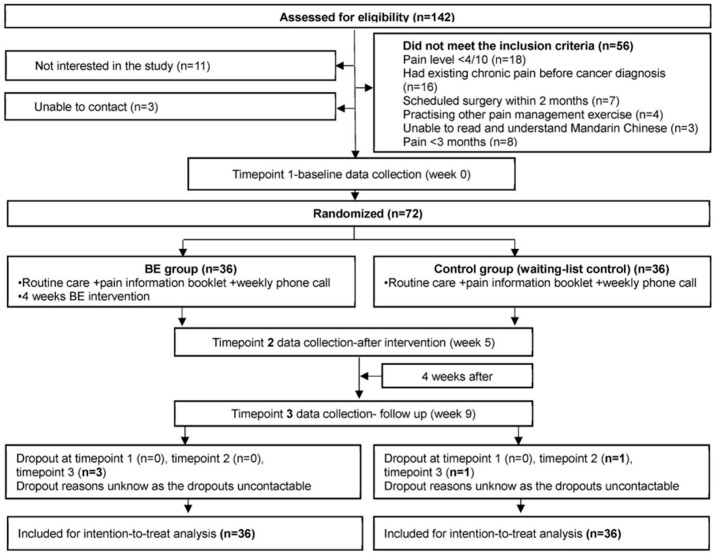
CONSORT flow diagram showing participant flow through the pilot randomised controlled trial, cited from Wang, Kwok [12]. BE = breathing exercise.

Figure 1 presents the CONSORT flow diagram showing the participant flow through all stages of the trial. Of the 142 individuals assessed for eligibility, 72 were randomised, and 68 completed all assessments through the 9-week follow-up period.

### 3.1. Feasibility Outcomes of the Pilot RCT

#### 3.1.1. Participants’ Baseline Demographic Characteristics

The mean age of participants was 52.8 years, with an average BMI of 23.1. Most were married (94.4%), non-religious (94.4%), and unemployed or retired (66.7%), with the majority reporting a low household income (monthly ≤¥6000) (70.8%). Nearly all were non-smokers and did not drink alcohol. Participants’ treatment histories, including the type of breast surgery and lymph node procedure, were collected at baseline. There were no significant differences between groups in surgical procedures or other treatment modalities, except for differences in current pain medication use and the expectations of BE effectiveness [12].

#### 3.1.2. Feasibility of the Subject Recruitment

A total of 142 patients were screened, of which 76 were referred by healthcare professionals (referral rate 54%). Among the screened patients, 86 met the inclusion criteria; 72 participated (recruitment rate 84%), and 68 completed the study (retention rate 94%). Four participants (6%) were lost to follow-up: three from the intervention group (all at Time point 3, week 9) and one from the control group (at Time points 2 and 3). The recruitment process took 19 weeks [12].

Four participants (5.6%) were lost to follow-up: three from the intervention group (all at Time point 3) and one from the control group (at Time points 2 and 3), yielding a retention rate of 94.4%. All three intervention group dropouts had completed the 4-week intervention and provided adherence data before being lost to follow-up at week 9. Three telephone contact attempts were made for each participant, but no responses were received. The complete participant flow is shown in Figure 1.

#### 3.1.3. Feasibility of the Study Questionnaires

The feasibility of the questionnaires was evaluated by examining missing values and completion time. No missing values were found for the HADS and QOLCSV-C at any time point (Time point 1: *n* = 72, Time point 2: *n* = 71, Time point 3: *n* = 68). The BPI had one missing value at Time point 3 (1.5% at the scale level), and the FACT-B showed missing responses for the item on satisfaction with sex life across all time points (Time point 1: 27.8%, Time point 2: 16.9%, Time point 3: 14.7%). The average completion times were 6.68 min for BPI, 6.12 min for HADS, 13.08 min for QOLCSV-C, and 9.01 min for FACT-B.

#### 3.1.4. Feasibility and Acceptability of the BE

##### The Adherence to the BE Intervention

**Overall adherence**: All 36 participants in the intervention group practiced the BE intervention, with an overall adherence rate of 82.41% (2492 total sessions completed ÷ 3024 minimum required sessions across all participants). This group-level metric reflects the overall intervention delivery and completion across the entire intervention group.

**Individual adherence:** Individual participant adherence rates ranged from 17.86% to 103.57%, with a median of 96.43% (IQR: 92.86–100.00%). Using established benchmarks [13], 58.3% (21/36) achieved an acceptable adherence (>80% of prescribed sessions) and 33.3% (12/36) achieved an optimal adherence (≥95% of prescribed sessions). The high median adherence and narrow interquartile range indicate that most participants successfully completed the prescribed intervention dose. Detailed individual adherence data by week are provided in Supplementary File S1 Table S1.

**Frequency and duration adherence:** All participants (100%, 36/36) reported completing 5 min sessions. Regarding frequency, 77.8% (28/36) followed the recommended minimum of three sessions per day, while 5.6% (2/36) exceeded this by practicing more than three sessions per day. For duration, 88.9% (32/36) adhered to the full 4-week intervention period (Table S2 in Supplementary file S1). Notably, only 27.8% (10/36) practiced exactly 7 days per week; however, many participants practiced 5–6 days per week with consistent session completion on practice days, contributing to the high overall adherence rate of 82.41%. Adherence was assessed via self-reported logbooks. The reasons for non-adherence are summarised in Table 1.

##### Sensitivity Analysis for Dropout

All analyses were conducted using an intention-to-treat approach. To assess the robustness of the feasibility findings given the unknown dropout reasons, a sensitivity analysis was conducted under a “worst-case scenario” assumption, whereby all four participants lost to follow-up discontinued due to study burden or dissatisfaction. Under this conservative assumption, retention remained at 94.4% (68/72), intervention adherence remained at 82.41% (all 36 intervention participants completed the intervention and provided adherence data), and questionnaire completion remained at 94.4%. All progression criteria continued to meet “Go (Green)” thresholds, demonstrating that the feasibility conclusions are robust regardless of dropout reasons.

##### Participants’ Feedback on the BE Intervention

All 36 participants in the intervention group provided feedback (100% response rate) after four weeks of the BE intervention period. The majority subjectively reported reduced anxiety, irritability, and pain, and improvements in mood, sleep, energy, and overall well-being on the self-report feedback questionnaires. These findings reflect participant perceptions of intervention acceptability and should not be interpreted as objective clinical outcomes. The majority found the dosage, frequency, and duration suitable, experienced few challenges, and were satisfied with the practice. Most participants indicated that they would continue the BE and recommend it to others (Table 2).

#### 3.1.5. Safety of the BE Intervention

Three participants (8.3%) reported mild, transient symptoms during the intervention period: light-headedness (*n* = 1), yawning (*n* = 1), and tiredness (*n* = 1). All symptoms were Grade 1 (mild), self-limiting, and resolved spontaneously without intervention. All were classified as possibly related to the breathing exercise intervention. Notably, these symptoms are consistent with the expected physiological responses to vagal nerve stimulation and parasympathetic nervous system activation, which is the proposed mechanism of action for breathing exercises. No serious adverse events occurred, and no participants discontinued the intervention due to adverse events. Table 3 summarises all reported adverse events.

#### 3.1.6. Progression Criteria Assessment

Our pilot trial outcomes met all “Go (Green)” progression criteria across key feasibility domains (Table 4), supporting progression to a fully powered RCT.

### 3.2. Findings from the Qualitative Process Evaluation

#### 3.2.1. Participants’ Demographic Characteristics

Twenty participants participated in the semi-structured interviews, including eleven from the intervention group and nine from the control group. The details of the participants’ characteristics are shown in Table 5.

#### 3.2.2. Theme and Subthemes

Two main themes and six subthemes emerged from the participants’ interviews.

Theme One: Experiences Related to Practising the BE.

(1) Subtheme One: Perceived the BE to be an easy and convenient exercise approach

Most participants perceived the BE as simple, flexible, and readily incorporated into daily routines. As one participant stated, “*It [the breathing exercise] is so simple and convenient. I can do it at home while watching TV or even after finishing dancing. It won’t take much time, so flexible….*” (P18, intervention group, 46 years, stage I breast cancer). The protocol was considered both acceptable and practical, with the breathing techniques described as easy to learn, manageable, and not time-consuming. Participants particularly valued the flexibility of practising the BE at home or during leisure activities, which facilitated a sense of autonomy in integrating the intervention into their daily lives.

(2) Subtheme Two: Challenges in adhering to the BE protocol

Participants identified forgetfulness, competing priorities, and daily routine variability as factors affecting adherence to the BE protocol, which is aligned with the RCT feasibility outcome assessment findings. For example, one interviewee highlighted, “*If I remember, I definitely do it, but I always forgot…you know, after the cancer treatment, I use a clock alarm to remind me to take medications, but it is impossible to set alarms for everything, only for those important ones*.” (P4, intervention group, 47 years, stage II breast cancer). Although they recognised its importance, maintaining consistent practice was challenging, often requiring memory cues or external reminders.

(3) Subtheme Three: Perceived benefits of the BE on pain relief and overall well-being

Most interviewees reported notable improvements in pain management and overall well-being from practising the BE, including reduced pain and numbness and enhanced relaxation. For instance, a participant expressed, “*I think [the] breathing exercise is good…um, since I started to practise [the] breathing exercise, my pain disappeared…I don’t have any pain, no pain at all. I had pain before, but I did [the] breathing exercise, [and] after I practised it for a while, the pain is gone now*.” (P10, intervention group, 48 years, stage II breast cancer). Additional benefits also included an improved mood, decreased depressive symptoms, and increased energy.

(4) Subtheme Four: Safe intervention, with only mild transient physical discomfort

Most interviewees reported no adverse effects from practising the BE. A few mild, transient discomforts, including tiredness, dizziness, and yawning, were noted, which led participants to pause or temporarily stop the practice. For example, as stated by a participant, “*[The] breathing exercise made me yawn. Once yawning is stopped, I usually continue [the] breathing exercise, no other problem or discomfort.*” (P17, intervention group, 58 years, stage II breast cancer). These discomforts generally resolved after a short rest, allowing participants to continue the BE.

Theme Two: Experiences Related to Study Participation and Procedures.

(1) Subtheme One: Sought potential health benefits by participating in the study

Participants were primarily motivated by the prospect of potential health benefits, including pain relief and improved overall well-being. Some were driven by curiosity and a desire to learn about the intervention, while others saw participation as a way to contribute to research and help others. For instance, a participant stated, “*I was thinking it would be good if my participation could help other people in the future.*” (P7, control group, 43 years, stage III breast cancer). Overall, there was a general sense of optimism and willingness to engage in the study.

(2) Subtheme Two: Questionnaires were easy to understand and not burdensome

The majority of interviewees indicated that the questionnaires were clear and easy to understand. They expressed satisfaction with both the length and content, reporting a positive experience with the data collection instruments, as stated by a participant “*I understand all the questions…all of them, no problem with understanding [the questions]. It didn’t take too long to complete.*” (P1, intervention group, 53 years, stage II breast cancer). Overall, the participants’ feedback reflected comfort and ease in completing the questionnaires, supporting the appropriateness and suitability of the research tools.

## 4. Discussion

This pilot study demonstrates a strong feasibility and acceptability for delivering a structured BE intervention in breast cancer survivorship care. The high recruitment and retention reflect a demand for non-pharmacological pain management options that are simple, flexible, and cost-free; qualitative feedback reinforced perceived convenience and autonomy. Collectively, these findings provide robust evidence supporting the conduct of a future full-scale RCT to evaluate effectiveness.

### 4.1. Feasibility of the Recruitment and Follow-Up Process

This study achieved high recruitment and retention rates, reflecting a strong demand for effective chronic pain management among breast cancer survivors. These positive outcomes may be attributed to the participants’ perceived benefits of the BE intervention, BE’s flexibility, and its safety. Both quantitative and qualitative data indicated a high participant satisfaction, with many expressing a willingness to continue the practice and recommend it to others.

Factors contributing to the high recruitment and retention rates might relate to the cost-free nature of BE. In this study, most participants lived in economically disadvantaged regions (monthly family income ≤ CNY ¥6000), and cancer survivors living in regional China experienced substantial financial strain due to cancer and its treatment [24]. Participation in a no-cost intervention with potential pain relief benefits may therefore have been appealing. A cultural familiarity with BE may also have contributed to recruitment success, as BEs are deeply rooted in Asian culture and widely accepted as a health-promoting practice [5]. Qualitative findings further supported this, with some participants citing cultural beliefs as a motivation to join the study. Additionally, the strong rapport established between participants and the research team, along with comprehensive preparatory activities, helped mitigate recruitment challenges and promote sustained engagement.

### 4.2. Feasibility of the Study Instruments

The study instruments were generally well accepted by the participants. A small number of missing responses were identified in item GS7 of the FACT-B, which pertains to sexual well-being. This pattern is commonly reported in Asian populations, where discussing sexual activity may be considered sensitive or embarrassing [25,26]. Similar findings have been documented among breast cancer survivors in Chinese [27,28], Indian [29], and Saudi Arabian [30] contexts.

Importantly, the FACT-B instrument was designed to accommodate such sensitivity. The original developers acknowledged the potential discomfort associated with this item and provided a clear scoring guidance that allows participants to omit the question without affecting subscale or total score calculations [31]. Therefore, despite the missing responses in item GS7, the validity of the FACT-B scores was not compromised. This finding highlights the importance of cultural sensitivity and transparent scoring procedures when using patient-reported outcome measures that include intimate or personal content.

### 4.3. Participants’ Adherence to the BE Intervention

The high adherence rates observed in this study suggest the strong feasibility and acceptability of the BE intervention protocol. These findings compare favourably with other self-administered mind–body interventions in cancer populations. For example, a systematic review of mindfulness-based interventions among cancer patients reported that the pool intervention adherence rate was 60% of the assigned amount across studies [32]. The flexibility of the protocol (3–5 sessions/day) may have contributed to sustained engagement, as participants could adapt the practice to their daily routines. Factors contributing to adherence may include participants’ perceived physical and psychological benefits, ease of practice, and the absence of financial cost. Qualitative data further supported these findings, with the participants describing perceived improvements in pain relief, energy, mood, and overall well-being. These subjective, self-reported experiences indicate a high intervention acceptability and support the feasibility for a definitive trial, though they do not constitute evidence of clinical effectiveness.

The most common challenge to adherence was that participants forgot to practice (30.6%). Although weekly reminder calls were made, these appeared insufficient to sustain daily practice. Future studies should explore more effective strategies to promote adherence, such as mobile reminders, visual prompts, or integrating BE into daily routines through habit rituals (e.g., practising BE after each meal) [33,34].

### 4.4. BE Intervention Safety 

Safety assessments indicated no serious adverse events associated with the BE intervention. Three participants (8.3%) reported mild, transient symptoms, light-headedness (*n* = 1), yawning (*n* = 1), and tiredness (*n* = 1), which all resolved spontaneously after a brief rest without the need for medical attention. The mild, transient symptoms reported are consistent with the known physiological effects of slow, deep BEs, which activate the parasympathetic nervous system via vagal nerve stimulation. These symptoms are generally considered indicators of successful autonomic modulation rather than harmful adverse effects. This activation is associated with a shift from a fight-or-flight state to a rest-and-digest state, a key mechanism in chronic pain management [35]. Nevertheless, we have reported them transparently as Grade 1 adverse events using the CTCAE Version 5.0 to maintain rigorous safety monitoring standards. The absence of serious adverse events and AE-related discontinuations supports the safety profile of this intervention. Overall, the BE intervention was well tolerated and considered safe for cancer survivors. Although BEs have been widely used in studies for hyperventilation [36], asthma [37], and chronic obstructive pulmonary disease [38], safety data are seldom reported [8,38]. Consistent with previous findings among cancer populations [39], this study reinforces the safety of BE and highlights the importance of systematically evaluating and reporting safety outcomes in future research to strengthen the evidence base for BE interventions.

## 5. Limitations and Future Directions

This pilot RCT was designed to evaluate feasibility and acceptability rather than clinical effectiveness. We acknowledge that this study used an open-label design and that outcome measures relied on self-reported questionnaires. Pain-related findings from participant feedback and qualitative interviews represent subjective perceptions appropriate for assessing intervention acceptability but not sufficient to establish clinical effectiveness; the planned definitive RCT will employ an adequate statistical power to evaluate efficacy. Future definitive trials should consider blinding strategies where feasible (e.g., attention-control or sham interventions) and use objective measurements as well to minimise bias in outcome assessment.

Several limitations should be acknowledged. First, our protocol did not pre-specify numerical progression criteria (Stop/Go/Amend thresholds) for advancing to a definitive trial. While we specified feasibility outcomes (recruitment, retention, adherence, safety, questionnaire completion), we did not establish a priori benchmarks for what would constitute “acceptable” feasibility. We therefore applied the progression criteria framework recommended by Mellor, Albury [16] post hoc to interpret our findings. Our pilot trial outcomes met all “Go (Green)” thresholds across key feasibility domains (see Table 4), supporting the progression to a definitive RCT. Second, the present study included only a single short-term follow-up at four weeks post-intervention, which limits our understanding of the long-term feasibility and sustainability of the intervention. We acknowledge that the adherence data were collected via self-reported logbooks verified during weekly telephone calls, which may be subject to recall and social desirability bias.

Several implications for future research and practice were identified. Future feasibility trials should pre-specify progression criteria at the protocol stage aligned with published frameworks [16] to enhance transparency and reduce interpretation bias. High recruitment, retention, and intervention adherence rates, together with satisfactory questionnaire completion and the absence of serious adverse events, support the feasibility, safety, and acceptability of the BE intervention and justify progression to a larger trial using similar procedures. The qualitative findings further reinforced the acceptability of the intervention and study processes, highlighting the value of participant-centred design features. Extended follow-up periods are recommended in future studies to assess the long-term adherence and sustainability of the practice. As BE is a cost-free, self-administered intervention and chronic pain imposes a substantial financial burden, future trials should also incorporate formal economic evaluations to assess cost-effectiveness and broader health system value.

## 6. Conclusions

The findings of this pilot RCT, complemented by the nested qualitative process evaluation, demonstrate the feasibility and acceptability of the BE protocol and trial procedures. These results justify progression to a larger, fully powered RCT to evaluate the effectiveness of the BE intervention for chronic pain management among breast cancer survivors.

## Figures and Tables

**Table 1:** Reasons for the participants’ non-adherence to the BE intervention protocol.

Reasons for Non-Adherence * to the BE	*n* = 26
Participant was unwell, stopped practising the BE	4 (11.1%)
Participant lost interest in practising the BE	2 (5.6%)
Participant dropped out of the study	4 (11.1%)
Participant’s pain disappeared, stopped practising the BE	1 (2.8%)
Participant forgot to practise the BE	11 (30.6%)
Participant was too busy to practise the BE	4 (11.1%)

Note: BE = breathing exercise. * = Non-adherence was defined as not meeting any of the prescribed practice criteria for the BE protocol (i.e., 5 min per session, three times per day, and seven days per week).

**Table 2:** Summary of the participants’ feedback on the BE intervention.

	BE Group (*n* = 36)/Number (%)				
Participants’ Feedback [I Feel…]	Absolutely Agree	Agree	No Opinion	Disagree	Absolutely Disagree
I am less anxious	6 (16.7%)	28 (77.8%)	0 (0.0%)	2 (5.6%)	0 (0.0%)
I am less irritable	5 (13.9%)	27 (75.0%)	0 (0.0%)	4 (11.1%)	0 (0.0%)
I am happier than before	3 (8.3%)	23 (63.9%)	0 (0.0%)	10 (27.8%)	0 (0.0%)
I have less pain	3 (8.3%)	29 (80.6%)	0 (0.0%)	4 (11.1%)	0 (0.0%)
My body is more relaxed than before	3 (8.3%)	28 (77.8%)	0 (0.0%)	5 (13.9%)	0 (0.0%)
My sleep is improved	1 (2.8%)	9 (25.0%)	0 (0.0%)	24(66.7%)	2 (5.6%)
I can do more activities than before	1 (2.8%)	4 (11.1%)	0 (0.0%)	29 (80.6%)	2 (5.6%)
I am more energetic	2 (5.6%)	16 (44.4%)	2 (5.6%)	16 (44.4%)	0 (0.0%)
My overall well-being is improved	2 (5.6%)	20 (55.6%)	2 (5.6%)	12 (33.3%)	0 (0.0%)
**Duration and frequency**	**Too long**		**Suitable**		**Too short**
Each session for 5 min	12 (33.3%)		24 (66.7%)		0 (0.0%)
3–5 sessions a day	6 (16.7%)		30 (83.3%)		0 (0.0%)
4 weeks’ duration	5 (13.9%)		31 (86.1%)		0 (0.0%)
**Challenges experienced**	**Never**		**Occasionally**		**Always**
Hard to learn the BE technique	35 (97.2%)		1 (2.8%)		0 (0.0%)
Too many interruptions, cannot concentrate on practising the BE	26 (72.2%)		6 (16.7%)		4 (11.1%)
Too tired to practise the BE	34 (94.4%)		1 (2.8%)		1 (2.8%)
Not interested in the BE	28 (77.8%)		7 (19.4%)		1 (2.8%)
**Satisfaction feedback**	**Very satisfied**	**Quite satisfied**	**Satisfied**	**Not satisfied**	**Very dissatisfied**
Were you satisfied with the BE?	0 (0.0%)	3 (8.3%)	33 (91.7%)	0 (0.0%)	0 (0.0%)
**Future practise and recommendations**	Absolutely will	Yes	Possibly will	No	Absolutely will not
Will you continue to practise the BE in the future?	2 (5.6%)	17 (47.2%)	14 (38.9%)	3 (8.3%)	0 (0.0%)
Would you recommend the BE to your friends?	3 (8.3%)	23 (63.9%)	9 (25.0%)	1 (2.8%)	0 (0.0%)

**Table 3:** Summary of adverse events.

Adverse Event	*n* (% of Intervention Group)	Severity Grade	Relatedness to Intervention	Resolution	Action Taken
Light-headedness	1 (2.8%)	Grade 1 (Mild)	Possibly related	Resolved spontaneously	None required
Yawning	1 (2.8%)	Grade 1 (Mild)	Possibly related	Resolved spontaneously	None required
Tiredness	1 (2.8%)	Grade 1 (Mild)	Possibly related	Resolved spontaneously	None required
Total participants with ≥1 AE	3 (8.3%)	-	-	-	-
Serious AEs	0 (0%)	-	-	-	-
AE-related discontinuations	0 (0%)	-	-	-	-

Note: Denominator = 36 participants in intervention group. All reported symptoms were transient and consistent with expected physiological responses to vagal nerve stimulation.

**Table 4:** Progression criteria assessment with context-specific benchmarks.

Feasibility Domain	Progression Criterion	Go (Green)	Pilot Trial Result	Decision
Recruitment rate	Proportion of eligible participants consented	≥70%	84% (72/86)	Go ✓
Retention rate	Proportion completing follow-up assessment	≥80% *	94% (68/72)	Go ✓
Adherence rate	Overall adherence to intervention protocol	≥70%	82.4% overall adherence	Go ✓
Data completeness	Valid outcome data obtained	≥90%	>95% across all measures	Go ✓
Safety	Serious adverse events and symptom burden	0 serious AEs; <15% mild symptoms	0 serious AEs; 8.3% mild symptoms	Go ✓

* Thabane, Ma [23] cite 80% as an example threshold for pilot study success. Note: Progression criteria developed post hoc following Mellor, Albury [16] framework. Benchmarks are context-specific and justified based on requirements for a viable definitive RCT.

**Table 5:** Characteristics of the interviewees (*n* = 20).

Demographic and Clinical Data		Number (%)
Study group (*n* = 20)	Intervention group	11 (55.0)
	Control group	9 (45.0)
Age (years) (*n* = 20)	30–39	1 (5.0)
	40–49	7 (35.0)
	50–59	9 (45.0)
	60–69	3 (15.0)
Education level (*n* = 20)	Primary school	14 (70.0)
	Junior high school	5 (25.0)
	Diploma	1 (5.0)
Marital status (*n* = 20)	Never married	0 (0.0)
	Married	19 (95.0)
	Divorced	0 (0.0)
	Widowed	1 (5.0)
Occupation (*n* = 20)	Professional worker	1 (5.0)
	Labourer	7 (35.0)
	Housewife	7 (35.0)
	Office clerk	1 (5.0)
	Others	4 (20.0)
Employment status (*n* = 20)	Employed	9 (45.0)
	Unemployed	10 (50.0)
	Retired	1 (5.0)
Religion (*n* = 20)	No religion	19 (95.0)
	Religious	1 (5.0)
Monthly household income (*n* = 20)	CNY ≤3000	3 (15.0)
	CNY 3001–6000	12 (60.0)
	CNY 6001–9000	3 (15.0)
	CNY >9000	2 (10.0)
Breast cancer stage (*n* = 20)	Stage I	5 (25.0)
	Stage II	13 (65.0)
	Stage IIIa	2 (10.0)
Average pain score at baseline (0–10) * (*n* = 20)	4–6	20 (100.0)
	≥7	0 (0.0)
Expectation for BE to relieve pain ^#^ (*n* = 20)	0–3	1 (5.0)
	4–6	4 (20.0)
	7–10	15 (75.0)
Pain relief after practising the BE reported by participants from the intervention group (*n* = 11)	Yes	10 (90.9)
	No	1 (9.1)
Satisfaction with practising the BE reported by participants from the intervention group (*n* = 11)	Very satisfied	1 (9.1)
	Satisfied	10 (90.9)

Note: CNY = Chinese Yuan; BE = breathing exercise. * = Pain intensity was rated on a 0–10 scale (0 = no pain; 10 = worst pain); ^#^ = expectation for BE to relive pain was assessed on a 0–10 scale (0 = minimum expectation; 10 = maximum expectation).

## Data Availability

The data presented in this study are available from the corresponding author upon reasonable request. The data are not publicly available due to privacy and ethical restrictions.

## Supplementary Materials

The following supporting information can be downloaded at: https://www.mdpi.com/article/10.3390/healthcare14050641/s1, File S1: Operational Procedures and Extended Methods for a Feasibility pilot RCT using Breathing Exercise Intervention for chronic pain management in Breast Cancer Survivors; Table S1: Individual and Overall BE Intervention Adherence in the Intervention Group (*n* = 36); Table S2: Summary of frequency and duration adherence. Refs. [4,7,12,13,17,20,40,41,42] are cited in the Supplementary Materials.
