# Supplementary material for: Breathing Exercise for Chronic Pain Management in Breast Cancer Survivors: Feasibility Outcomes and Qualitative Insights from a Pilot Randomised Controlled Trial
Source: Healthcare (Basel). 2026 Mar 3;14(5):641. doi: 10.3390/healthcare14050641 (PMC12985228; doi:10.3390/healthcare14050641)
Supplement: Supplementary file 1 [file healthcare-14-00641-s001.zip › healthcare-4161447-supplementary.pdf]

Supplementary File:

Supplementary File S1: Operational Procedures and Extended Methods for a Feasibility pilot RCT using Breathing Exercise Intervention for chronic pain management in Breast Cancer Survivors

1. Trial Overview and Rationale

Project Overview: This study was conducted in accordance with the MRC Framework for Developing and Evaluating Complex Interventions (Craig et al., 2008; Skivington et al., 2021). It comprised two sequential phases. Phase I focused on the breathing exercise (BE) intervention development that is published in Wang et al. (2023b). Phase II involved a pilot RCT with an embedded qualitative process evaluation to assess the feasibility and acceptability of the BE intervention and research procedures, details can be found in prior publications (Wang et al., 2024; Wang et al., 2022). The study was prospectively registered with ClinicalTrials.gov (ID: NCT05257876, date: February 3, 2022). The present manuscript focusing on reporting pilot RCT feasibility outcomes only, and does not include any hypothesis testing or between-group comparisons of intervention efficacy.

Pilot RCT Design: Open-label pilot RCT with a nested qualitative process evaluation.

Randomisation: Independent online randomisation 1:1 with permuted block sizes (2, 4, 6).

Participants: Participants' treatment histories, including type of breast surgery and lymph node procedure (ALND or SLNB) were collected at baseline. There were no significant differences between groups in surgical procedures or other treatment modalities, as pre-piously reported in Wang et al. (Wang et al., 2024)(Wang et al., 2024)

Allocation Concealment: Concealment. Allocation provided only after enrolment; record keeping procedures stored in the trial master file.

Feasibility objective: Per the MRC Framework and CONSORT extension for pilot/feasibility studies, quantitative analyses were descriptive only; no hypothesis testing or between-group efficacy comparisons were undertaken.

2. Standard (Routine) Care Protocol

To aid replicability, the following components of "standard care" were used alongside the BE protocol:

- Oncology follow-up and symptom management according to institutional protocols.
- Referral to rehabilitation services and psychosocial support as required.

3. Breathing Exercise (BE) Intervention

Technique: Slow pursed-lip breathing with inspiration-to-expiration ratio 1:2–3.

Dosage: 3–5 sessions/day, 5 minutes/session, for 4 weeks.

Training & Competency: Nurse-delivered, face-to-face instruction supported by a demonstration video; participants demonstrated technique proficiency prior to independent practice.

Contraindications (screening): Exclude severe physical limitations preventing BE practice and other factors listed in eligibility/exclusion criteria.

Safety Monitoring: Weekly phone calls to monitor discomfort/adverse events; daily logbooks to record sessions and any symptoms. Mild transient effects (e.g., light-headedness, yawning, tiredness) were self-limiting and required no treatment.
Control Offer: Post-trial BE program was offered to control group participants after final data collection.

4. Feasibility assessment

Referral rate: the proportion of referrals made by clinicians relative to all referrals received from inpatient and outpatient clinics;
Recruitment rate: the proportion of eligible individuals who consented to participate in the study;
Retention rate: the proportion of participants who completed the study through to follow-up assessment;
Dropout rate: the proportion of participants who discontinued participation after randomisation;
Time required to recruit the target sample size;
Feedback from participants who withdrew, including their reasons for discontinuation.
Qualitative insights: data was collected through semi-structured interviews of participants from the pilot RCT about their experiences of participating in the pilot RCT study and practicing BE.
Safety: data was collected through daily logbooks and weekly phone calls about any side effects of practicing BE.

5: Feasibility of study questionnaires

BPI: Brief Pain Inventory,
HADS: Hospital Anxiety and Depression Scale,
QOL-CSV: Quality of Life Cancer Survivors Version,
FACT-B: Functional Assessment of Cancer Therapy–Breast.

6: Data collection time points:

Baseline (Time point 1, week 0),
post-intervention (Time point 2, week 5),
Follow-up (Time point 3, week 9).

7. Adherence Monitoring & Calculation

Prescribed sessions: Minimum **84 sessions** over 4 weeks (3/day × 7 days × 4 weeks).
Recording tools:

- **Daily logbooks** (self-report; session count, timing, any symptoms).
- **Weekly phone calls** (adherence support; side-effect check; changes in treatment).

Adherence benchmark:

- **Acceptable:** >80% of prescribed sessions.
- **Optimal:** ≥95% of prescribed sessions. (Benchmark reference: Wahl et al., 2005.)

Formula: Adherence (%) = (Total BE sessions completed / 84) × 100
Common reasons for non-adherence (for planning future trials): Forgetfulness, being too busy, temporary illness, loss of interest; consider reminder systems and habit-linking (e.g., after meals).

Table S1. Individual and Overall BE Intervention Adherence in the Intervention Group (n=36)

Participants in the Intervention Group	No. of BE Sessions Practised Week 1	No. of BE Sessions Practised Week 2	No. of BE Sessions Practised Week 3	No. of BE Sessions Practised Week 4	Total No. of Sessions Practised in Four Weeks	Minimum No. of Sessions Required	Adherence Rate (%)
1	12	15	21	12	60	84	71.43
2	21	28	21	21	91	84	108.33
3	18	18	12	14	62	84	73.81
4	21	21	18	21	81	84	96.43
5	18	12	15	21	66	84	78.57
6	9	9	9	9	36	84	42.86
7	12	15	0	21	48	84	57.14
8	15	15	15	15	60	84	71.43
9	15	18	21	21	75	84	89.29
10	10	15	21	21	67	84	79.76
11	15	15	21	21	72	84	85.71
12	21	21	21	21	84	84	100.00
13	15	15	18	21	69	84	82.14
14	21	21	21	21	84	84	100.00
15	12	15	18	21	66	84	78.57
16	18	12	15	21	66	84	78.57
17	15	18	21	21	75	84	89.29
18	15	0	0	0	15	84	17.86
19	21	21	21	21	84	84	100.00
20	21	21	21	28	91	84	108.33
21	15	12	21	21	69	84	82.14
22	21	21	21	21	84	84	100.00
23	21	18	21	21	81	84	96.43
24	15	21	21	21	78	84	92.86
25	18	21	21	0	60	84	71.43
26	15	21	21	21	78	84	92.86
27	15	15	21	21	72	84	85.71
28	12	15	15	12	54	84	64.29
29	15	12	15	18	60	84	71.43
30	21	21	21	21	84	84	100.00
31	21	21	21	15	78	84	92.86
32	21	21	6	0	48	84	57.14

33	21	21	21	21	84	84	100.00
34	21	21	21	21	84	84	100.00
35	21	21	0	0	42	84	50.00
36	21	21	21	21	84	84	100.00
**Mean (SD)**	**18.1 (3.9)**	**18.8 (5.0)**	**17.8 (7.0)**	**18.4 (6.8)**	**69.2 (16.4)**	**84 (0)**	**82.41 (20.5)**
**Median (IQR)**	**21 (15-21)**	**21 (15-21)**	**21 (15-21)**	**21 (21-21)**	**75 (66-84)**	**84 (84-84)**	**89.29 (78.57-100.00)**
**Range**	**9-21**	**0-28**	**0-21**	**0-28**	**15-91**	**84**	**17.86-108.33**
**Total**	**651**	**677**	**640**	**662**	**2,492**	**3,024**	**82.41%**

Note: BE = breathing exercise. Adherence rate calculated as (total sessions completed by each participant) ÷ (84 minimum required sessions) × 100. Overall group adherence rate = 2,492 total sessions completed ÷ 3,024 required sessions = 82.41%. Some participants exceeded the minimum requirement of 3 sessions/day, resulting in adherence rates >100%. Three participants (Participants 18, 32, 35) had adherence rates <60% but remained in the study and completed all assessments, demonstrating the intention-to-treat approach.

Table S2. Summary of frequency and duration adherence

**Adherence to the Frequency and Duration of the BE Intervention Protocol**	**Intervention Group (n=36) Number (%)**
**Frequency of practising the BE**	
<3 times a day	6 (16.7%)
3 times a day (standard)	28 (77.8%)
>3 times a	2 (5.6%)
**Days per week practising the BE intervention**	
<7 days a week	26 (72.2 %)
7 days a week (standard)	10 (27.8%)
**Duration of practising the BE**	
5 minutes per session	36 (100.0%)
<4 weeks	4 (11.1%)
4 weeks (standard)	32 (88.9%)
**Adherence to the BE intervention protocol (84 sessions are required over 4 weeks)***	
Acceptable (>80%)	21 (58.3%)
Optimal (≥95%)	12 (33.3%)

Note: BE=breathing exercise; *=based on the adherence benchmark that >80% is considered acceptable and ≥95% is considered optimal(Wahl et al., 2005).

8. Questionnaire Administration & Missing-Data Handling

Administration mode: Baseline in person; subsequent assessments face-to-face or by telephone per participant preference.

Missing data calculation: Item-level missing data: referred to the percentage of unanswered items; **Scale-level missing data:** refer to the percentage of questionnaires with at least one missing item.

Handling sensitive items (FACT-B GS7). The FACT-B manual allows omission of the sexual well-being item without compromising subscale/total scores; apply the manual's scoring guidance when GS7 is missing.

Use in main text: Keep the instrument list and timepoints; move the detailed **missing-data definitions**, **mode of delivery**, and

FACT-B scoring manual note: per FACT-B Scoring Guidelines (Version 4), it is said:

"Instructions:

1. Record answers in "item response" column. If missing, mark with an X

2. Perform reversals as indicated, and sum individual items to obtain a score.

3. Multiply the sum of the item scores by the number of items in the subscale, then divide by the number of items answered. This produces the subscale score.

4. Add subscale scores to derive total scores (TOI, FACT-G & FACT-B).

5. The higher the score, the better the QOL." (FACIT.org, 2025, p. 1)

The missing items in the study were handled based on the guidelines accordingly.

9. Ethics and Data Governance

Approvals. Charles Darwin University (H21089) and Affiliated Hospital of Southwest Medical University (KY2022107).

Consent & confidentiality. Written informed consent; secure storage of recordings/transcripts.

Data availability. Available from corresponding author upon reasonable request; not publicly shared due to privacy/ethical restrictions.

10. Daily BE logbook (template)

Date:

Session 1 (time): ___ Completed: Yes/No Symptoms: […]

Session 2 (time): ___ Completed: Yes/No Symptoms: [...]

Session 3 (time): ___ Completed: Yes/No Symptoms: [...]

Optional Session 4/5:

Notes (interruptions, reasons for missing):

11. Weekly call checklist (brief script)

1) Confirm sessions completed since last call.
2) Ask about any discomfort/adverse events.
3) Record reasons for any missed sessions.
4) Encourage adherence; agree on reminder strategy for the coming week.
5) Note any changes in medical treatment.

12. Qualitative process evaluation details

Qualitative Methods: Interview Guide, Training & Analysis
Sampling: Purposive sampling to capture variation in (a) baseline expectations of BE effectiveness (high/low), (b) perceived pain relief after 4 weeks (yes/no), and (c) satisfaction (yes/no). Sample size guided by data saturation. In total 20 participants included, 11 from intervention group and 9 from the control group.
Data collection: Semi-structured interviews, audio-recorded with consent; transcribed verbatim in Chinese; accuracy checked by interviewees and team.
Analysis: Thematic analysis in **NVivo 14** using Braun & Clarke's six-step approach (familiarisation, coding, theme searching, reviewing, defining/naming, reporting). Consensus achieved through cross-checking and iterative discussion.
Interviewer training (five phases): Ethics; qualitative methods; mock interviews; fidelity checks; supervised practice—structured program to ensure data quality and consistency
Trustworthiness: Confirmability (member checking), credibility (careful design and iterative discussions), transferability (detailed procedures), and dependability (transparent reporting) (Nowell et al., 2017).

Thematic analysis steps in qualitative process evaluation (NVivo 14)
Step 1: Familiarisation with transcripts (Chinese; verbatim).
Step 2: Generate initial codes (researcher; cross-check with team).
Step 3: Search for themes (group codes; draft thematic map).
Step 4: Review themes (refine with team; ensure coherence).
Step 5: Define and name themes (final labels; exemplar quotes).
Step 6: Report (theme description; trustworthiness notes).
More details can be found in the thesis (Wang, 2025).

Qualitative process evaluation findings

Two main themes and six subthemes emerged from the participants' interviews.
Theme One: Experiences related to practising the BE
(1) Subtheme One: Perceived the BE to be an easy and convenient exercise approach
Most participants perceived the BE as simple, flexible, and readily incorporated into daily routines. As one participant stated, *"It [the breathing exercise] is so simple and convenient. I can do it at home while watching TV or even after finishing dancing. It won't take much time, so flexible…."* The protocol was considered both acceptable and practical, with the breathing techniques described as easy to learn, manageable, and not time-consuming Participants particularly valued the flexibility of practising the BE at home or during leisure activities, which facilitated a sense of autonomy in integrating the intervention into their daily lives.
(2) Subtheme Two: Challenges in adhering to the BE protocol
Participants identified forgetfulness, competing priorities, and daily routine variability as factors affecting adherence to the BE protocol, which is aligned with the RCT feasibility outcome assessment findings. For example, one interviewee highlighted, *"If I remember, I definitely do it, but I always forgot…you know, after the cancer treatment, I use a clock alarm to remind me to take medications, but it is impossible to set alarms for everything, only for those important ones*." Although they

recognised its importance, maintaining consistent practice was challenging, often requiring memory cues or external reminders.

(3) Subtheme Three: Positive impact of the BE on pain relief and overall well-being

Most interviewees reported notable improvements in pain management and overall well-being from practising the BE, including reduced pain and numbness and enhanced relaxation. For instance, a participant expressed *"I think [the] breathing exercise is good…um, since I started to practise [the] breathing exercise, my pain disappeared…I don't have any pain, no pain at all. I had pain before, but I did [the] breathing exercise, [and] after I practised it for a while, the pain is gone now*." Additional benefits also included improved mood, decreased depressive symptoms, and increased energy.

(4) Subtheme Four: Safe intervention, with only mild transient physical discomfort

Most interviewees reported no adverse effects from practising the BE. A few mild, transient discomforts, including tiredness, dizziness, and yawning, were noted, which led participants to pause or temporarily stop the practice. For example, stated by a participant, *"[The] breathing exercise made me yawn. Once yawning is stopped, I usually continue [the] breathing exercise, no other problem or discomfort."* These discomforts generally resolved after a short rest, allowing participants to continue the BE.

Theme Two: Experiences related to study participation and procedures

(1) Subtheme One: Sought potential health benefits by participating in the study

Participants were primarily motivated by the prospect of potential health benefits, including pain relief and improved overall well-being. Some were driven by curiosity and a desire to learn about the intervention, while others saw participation as a way to contribute to research and help others. For instance, a participant stated, *"I was thinking it would be good if my participation could help other people in the future."* Overall, there was a general sense of optimism and willingness to engage in the study.

(2) Subtheme Two: Questionnaires were easy to understand and not burdensome

The majority of interviewees indicated that the questionnaires were clear and easy to understand. They expressed satisfaction with both the length and content, reporting a positive experience with the data collection instruments. An example from a participant, *"I understand all the questions…all of them, no problem with understanding [the questions]. It didn't take too long to complete."* Overall, participants' feedback reflected comfort and ease in completing the questionnaires, supporting the appropriateness and suitability of the research tools.

References

Craig, P., Dieppe, P., Macintyre, S., Michie, S., Nazareth, I., & Petticrew, M. (2008). Developing and evaluating complex interventions: the new Medical Research Council guidance. *bmj*, *337*.

FACIT.org. (2025). *FACT-B Scoring Downloads*. Retrieved 18 Dec. from https://www.facit.org/measures-scoring-downloads/fact-b-scoring-downloads

Nowell, L. S., Norris, J. M., White, D. E., & Moules, N. J. (2017). Thematic analysis: Striving to meet the trustworthiness criteria. *International journal of qualitative methods*, *16*(1), 1609406917733847.

Skivington, K., Matthews, L., Simpson, S. A., Craig, P., Baird, J., Blazeby, J. M., Boyd, K. A., Craig, N., French, D. P., & McIntosh, E. (2021). A new framework for developing

and evaluating complex interventions: update of Medical Research Council guidance. *bmj*, *374*.

Wahl, C., Gregoire, J.-P., Teo, K., Beaulieu, M., Labelle, S., Leduc, B., Cochrane, B., Lapointe, L., & Montague, T. (2005). Concordance, compliance and adherence in healthcare: closing gaps and improving outcomes. *Healthc Q*, *8*(1), 65-70.

Wang, H. (2025). *An evidence-based breathing exercise intervention for chronic pain management in breast cancer survivors: A phase II randomised controlled trial* (Doctoral dissertation, Charles Darwin University). Charles Darwin University Research Portal. https://researchers.cdu.edu.au/en/studentTheses/an-evidence-based-breathing-exercise-intervention-for-chronic-pai/

Wang, H., Kwok, W. H., Yao, L.-Q., Liu, X.-L., Bressington, D., Chen, M.-L., Huang, H.-Q., Wang, T., & Tan, J.-Y. B. (2024). An evidence-based breathing exercise intervention for chronic pain management in breast cancer survivors: A phase II randomized controlled trial. *European Journal of Oncology Nursing*, *71*, 102625.

Wang, H., Tan, J.-Y. B., Wang, T., Liu, X.-L., Bressington, D., Zheng, S.-L., & Huang, H.-Q. (2022). Feasibility and potential effects of breathing exercise for chronic pain management in breast cancer survivors: study protocol of a phase II randomised controlled trial. *BMJ Open*, *12*(12), e064358.

Wang, H., Wang, T., Tan, J.-Y. B., Bressington, D., Zheng, S.-L., Liu, X.-L., & Huang, H.-Q. (2023b). Development and validation of an evidence-based breathing exercise intervention protocol for chronic pain management in breast cancer survivors. *Pain Management Nursing*, *24*(3), 357-364.
